# Titanium Implants Coated with a Bifunctional Molecule with Antimicrobic Activity: A Rabbit Study

**DOI:** 10.3390/ma13163613

**Published:** 2020-08-15

**Authors:** Antonio Scarano, Francesco Carinci, Tiziana Orsini, Luca Valbonetti, Erda Qorri, Carlo Alberto Bignozzi, Felice Lorusso

**Affiliations:** 1 Department of Medical, Oral and Biotechnological Sciences, University of Chieti-Pescara, Via dei Vestini, 31 66100 Chieti, Italy; drlorussofelice@gmail.com; 2 Department of Maxillofacial Surgery, University of Ferrara, Via Savonarola, 9, 44121 Ferrara FE, Italy; francesco.carinci@unife.it; 3 CNR—National Research Council, Institute of Cell Biology and Neurobiology (IBCN), Via Ercole Ramarini 32, 00015 Monterotondo RM, Italy; tiziana.orsini@cnr.it; 4 Unit of Basic and Applied Biosciences, Faculty of Veterinary Medicine, University of Teramo, SP18, 64100 Teramo TE, Italy; lvalbonetti@unite.it; 5 Faculty of Medical Sciences, Albanian University, Bulevardi Zogu 1, 1031 Tirane, Albania; erda79@yahoo.com; 6 Department of Chemistry, University of Ferrara, Via Savonarola, 9, 44121 Ferrara FE, Italy; g4s@unife.it

**Keywords:** bifunctional molecule, antimicrobic activity, anatase, dental implants, osseointegration, implant surface

## Abstract

Background: Various surface treatments have been tested for titanium implants aiming at increasing their surface biocompatibility and their biological characteristics, but also the efficiency of the implant surface will have to be improved to drastically decrease peri-implantite and mucosite. In fact, the peri-implantitis and peri-implant mucositis have a high incidence in clinical practice. The nanofabrication techniques that offer the possibility to achieve the implant surface that reduces bacterial colonization could influence the osteointegration. The aim of this research was to evaluate the bone response to titanium implants coated with a bifunctional molecule with antimicrobic activity consisting of a combination of silver ions covalently bound to titanium dioxide nanoparticles. Methods: A total of 36 implants were inserted into 18 older New Zealand white male rabbits. They had two different surfaces. The implants Control group was characterized by an acid-etched and sandblasted surface treatment, and the Test implants had an acid-etched and sandblasted surface coated with a silver ion covalently bound to titanium dioxide nanoparticles in the solution. Results: No statistically significant difference of the bone density was evidenced between Control and Test implants at two weeks (*p*-value = 0.623), four weeks (*p*-value = 0.339), and eight weeks (*p*-value = 0.461). Moreover, no statistically significant difference of the bone-implant contact percentage was evidenced between Control and Test implants at two weeks (*p*-value = 0.938), four weeks (*p*-value = 0.307), and eight weeks (*p*-value = 0.294). The effectiveness of the present investigation demonstrated no adverse effects on osseointegration, and no statistically significant differences were observed in the bone density and percentage of bone-implant contact between Test and Control implants at all the experimental time points (two, four, and eight weeks). Conclusions: Titanium implants coated with the silver-anatase solution bind very well to the bone and did not have an adverse effect on the bone tissue in a rabbit model. These facts suggest possible clinical applications for the silver composition.

## 1. Introduction

Bone formation in direct contact with the surface of a dental implant is the result of biological events due to differentiation and proliferation of pre-osteoblasts into osteoblasts cells, producing an osteoid matrix and mineralization with subsequent formation of the bone-implant interface. These intricate biological phenomena form with direct contact at the level of the interface between bone tissue and the implant surface. The quality and quantity of the bone-implant interface is strongly influenced by the micro [1] and macro morphology of the surfaces [2]. Different techniques have been used for surface modification such as coating techniques, chemical alteration of surfaces, anodization techniques, and mechanical ones. Another strategy to improve bone implant contact is the binding on the Ti surface with peptides, enzymes, and other main molecular components of the extracellular matrix, such as type I collagen to increase the adhesion of the osteoblasts [3]. Titanium biocompatibility is directly connected to the properties of its oxide layer surface, including its structure, composition, and morphology [4]. Initial events that take place after implant placement are in part determined by biomolecules that interact with the implant surface [5]. The emerging trend of surface modifications of dental implants involves attempts at controlling surface chemistry [6]. Surface chemistry seems certainly to be a key factor for the improvement of osseointegration [6]. Various surface treatments have been tested for titanium implants aiming at increasing their surface biocompatibility and their biological characteristics [7,8]. However, today there is a great interest in new fabrication techniques of coating for a dental implant that reduces bacterial colonization and offers the possibility for improving dental implants survival. The bacterial biofilm formation on the dental implant could induce an inflammatory phenomenon and generate an increased risk for peri-implantitis, mucositis, and bone loss. In fact, the peri-implantitis and peri-implant mucositis have a high incidence in clinical practice. Anatase (TiO_2_) and silver ions were used against bacterial adhesion on the dental implant, healing screw, and abutment.

Titanium dioxide, being a versatile and resistant material, has been implemented in different industrial uses, for example, those for solar energy and photocatalysis [9], food coloring, cosmetics (nolan), ceramics, sunscreens, and toothpaste [9]. There are three different crystal lattices of titanium dioxide: Anatase, rutile, and brookite [10]. Normally, a random allocation of two titanium oxides (anatase and rutile) is present on the titanium surface, which is responsible for the its particular properties [11]. Rutile is believed to be the stable form, while anatase is metastable and changes to rutile at high temperatures [12]. Anatase shares almost the same characteristic as rutile in terms of luster, hardness, and density [13]. Both forms are characterized by the same symmetry, but they differ slightly in crystal habit and, more distinctly, in cleavage [13]. Anatase shows stronger interactions among the metal and support [7,8] and has been shown to be able, in vitro studies, to absorb more OH^−^ and PO43^−^ than rutile, in biological fluids, which favors the deposition of a bone-like apatite formation [4,7,8]. Anatase can be used as a colloidal solution and, in such a way, it is possible to change the implant surface properties, with no modification of the properties determined by the surface macro, micro, and nano design [4,12]. The bulk properties of the material remain while only the outermost part of the surface in contact with the tissues is modified [7,8]. Various systems can be employed to produce an homogeneous anatase coating around the implant surface [4]. This coating endows the implant surface with special characteristics: Some of which have some genetic effects on the osteoblasts [4,7,8]. UV irradiation causes the anatase coating to release free radicals, such as H_2_O_2_, OH, O_2_^−^, and HO_2_^−^. The lysis of bacteria and other organic substances is a result of this potent oxidizing power [14,15,16,17]. There have been several studies on the bactericidal properties of TiO_2_ in relation to organisms such as Escherichia coli [18,19]. The exact bactericidal mechanism is not yet fully understood [15,18]. This result may be due to the elimination of the protecting cell wall of the bacteria, leading to an augmentation in the cell permeability which determines a loss of its intracellular contents, leading to cell death [15,18]. Moreover, silver ions have good antimicrobial activity and have been used to prevent infections. Silver ions were used as a constituent of coatings on medical implants and medical devices. Our previous study showed that with this coating it is possible to increase the transcription of some miRNAs which enhance osteoblast activity with no major adverse effect on the cells [4]. However, this coating that offers the possibility to achieve the implant surface that reduces bacterial colonization could influence the osteointegration. The first null hypothesis resulted in there being no adverse effect on the bone healing and no statistically significant differences in osseointegration and cell responses in the implant bone contact and bone density between anatase-silver and uncoated implants. The aim of this research was to evaluate the bone response to titanium implants coated with a bifunctional molecule with antimicrobic activity consisting of a combination of silver ions covalently bound to titanium dioxide nanoparticles.

## 2. Materials and Methods

### 2.1. Preparation of the Anatase-Silver Coating

General methods for the preparation of the anatase-silver coating have been previously reported in two patents: (WO 2007/122651-FUNCTIONAL NANOMATERIALS WITH ANTIBACTERIAL AND ANTIVIRAL ACTIVITY 01.11.2007 B22F 1/02 PCT/IT2006/000280 and WO 2008/020460-NANOMATERIAL COATINGS FOR OSTEOINTEGRATED BIOMEDICAL PROSTHESES 21.02.2008 A61L 27/30 PCT/IT2006/000450).

The preparations have been done according to the following procedures:

#### 2.1.1. Preparation of the Suspension of Titanium Dioxide Nanoparticles

An amount of 300 mL of distilled water and 2.1 mL of 65% nitric acid (Aldrich, St. Louis, MO, USA) were mixed in a beaker, and 5 mL of titanium isopropoxide (Fluka) was slowly added dropwise over 10 min. A white precipitate of titanium dioxide was observed to form and the suspension was heated at 80 °C under stirring for 12 h. The colloidal suspension was allowed to concentrate during the heating process to a final volume of ca 150 mL corresponding to a TiO_2_ concentration of 100 g/L. At the end of this process the diameter of the titanium dioxide nanoparticles was in the range 6–15 nm. The suspension which had been concentrated to 100 mL was finally diluted by the addition of 400 mL of distilled water and 500 mL of 99% ethanol, giving rise to a final transparent solution containing 1% of TiO_2_ at pH 2.

#### 2.1.2. Functionalization of Titanium Dioxide Nanoparticles with Silver Ions

A 0.05 g amount of mercaptophenylboronic acid ligand (MFB) (Aldrich) was added under stirring to 100 mL of the suspension of TiO_2_ nanoparticles. Stirring was continued for 24 h at room temperature and a 0.06 g amount of silver lactate (Aldrich) was finally added. With this procedure, the terminal boronic acid of the bifunctional MFB ligand binds the titanium dioxide surface allowing the sulphur of the mercapto moiety to coordinate a silver ion.

##### Implants Coating

The antimicrobial suspension described in Section 2.1.2 was applied by spray coating to the implants, followed by drying at 200 °C for 10 min in a ventilated oven. The implants were finally packed and sterilized by γ rays.

### 2.2. Scanning Electron Microscopy Observations (SEM)

The implant surface characterization of both the Control and Test group was performed by the Scanning Electron Microscope (SEM) (VEGA LSH TESCAN-Tescan Sro, Brno, Czech Republic). The evaluation of the surface roughness was performed by the Alicona Mex 5.0 H1 imaging software package (Alicona Imaging GmbH, Grambach, Graz, Austria).

On all samples, four different areas were evaluated at a scan rate of 0.1 Hz. Moreover, the surface microstructure parameters were evaluated by the use of a dedicated software package. For the measurement of the surface roughness, the different parameters were recorded for each of the ten implants: Means for each parameter were calculated and average roughness—Ra, root-mean-square roughness of profile—Rq, maximum peak to valley height of roughness profile—Rt, mean peak to valley height of roughness profile—Rz, maximum peak to valley height of roughness profile within a sampling length—Rmax, as well as the standard deviation are shown in Table 1.

### 2.3. Animals and Surgical Procedure

**Figure 1:**
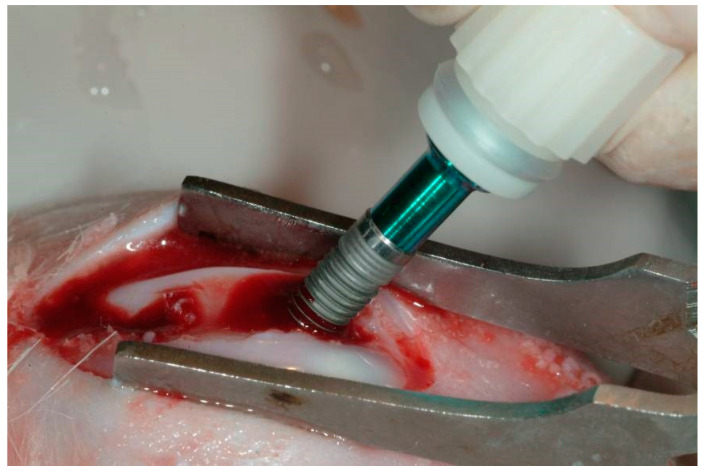
Insertion of the implant in a rabbit knee joint.

A total of eighteen adult New Zealand male rabbits, characterized by a weight of about 2.6 kg, were treated in the present investigation. The research was approved by the local Ethics Committee of Albania University and carried out in accordance with the relevant guidelines and regulations of Italian law animal research. A total of 36 implants for both surfaces (18 implants with acid-etched and sandblasted surfaces were used as the Control; and 18 implant acid-etched and sandblasted surfaces coated by the anatase-silver coating solution were used as the Test) (blasted surface—BWS) (Dental Tech, Misinto, Italy). Moreover, the acid etching treatment was performed by the use of a mixture of fluoridric and nitric acid, while the sandblasting treatment was provided by 60–120 µm Al_2_O_3_ particles. The implants were made of titanium grade 5, had a dimension of 4.5 mm × 13 mm, and were produced with high precision instruments. The implants were inserted, one Control into the left articular femoral knee-joint and one Test into the right articular femoral knee-joint according to a previously described technique. Each rabbit received one Control and one Test implant, one in each joint. In all animals, the anesthesia was administered before the surgery procedure with an intramuscular infiltration of diazepam (1.5 mg/kg bwt) and fluanizone (0.6 mg/kg bwt). The local anesthesia was administered by the use of 1 mL of a 2% lidocain/adrenalin solution. To access the articular joint bone, an incision with a periosteal flap was performed. A conventional dental handpiece with a physio-dispenser (Vario-Surgery NSK, Tochigi, Japan) was used for implant bed preparation. The preparation of the bone implant osteotomy was performed with drills cooled by a generous saline irrigation (Figure 1).

During the experimental study, a total of two rabbits died and these animals were substituted. The rabbits were euthanized at 15, 30, and 60 days, by an intravenous overdose of Tanax. A total of 36 dental implants were retrieved in the present investigation. The implants and surrounding tissues retrieved were stored immediately in a solution of 10% buffered formalin and processed in order to produce thin ground sections. The samples collected were processed by the use of the Precise 1 Automated System (Assing, Rome, Italy) [20]. Then, the samples were subjected to a dehydration process by a graded ethanol rinse and subsequently embedded into a glycolmethacrylate resin (Technovit 7200 VLC, Kulzer, Wehrheim, Germany). At the end of the polymerization procedure, the samples were sectioned, along the longitudinal profile of the dental implant by the use of a high-precision diamond disc at about 150 mm and ground down to about 30 mm by a dedicated grinding machine. A total of three slides were obtained from each sample and they were stained by toluidine blue and acid fuchsin. The slides were then observed in normal transmitted light by a Nikon microscope ECLIPSE (Nikon, Tokyo, Japan). The aspects of newly formed and mature bone could be classified in consequence to the histological color of the tissues (light red = old matrix, dark red = new matrix) and their quantity was expressed in percentage (mean ± SD).

The values of bone implant contact (BIC), bone area inner threads (BAIT), and bone area outer threads (BAOT) were measured to investigate the osteogenic parameters at the level of the dental implant surface and the measurements were expressed in percentage (mean ± SD). The BAIT in direct proximity to the implant surface was measured within the thread region, while the BAOT values were calculated distantly from the fixture interface and extended for the same size into the adjacent new/old bone The values of BIC, BAIT, and BAOT were calculated under light microscopy observation by a high-resolution video camera (16.25-megapixel) (Digital Sight series microscope cameras), a high definition monitor, and a computer workstation (Notebook Toshiba Satellite pro r50-c-15w, Minato, Japan). The optical system was connected to a dedicated histometry software package for image capturing, registered by the use of a Sony α330 digital camera (Sony, Minato, Japan), and a morphometric analysis by a digital image-analysis was performed (NIS-Elements AR 3.0 software, Nikon, Minato, Japan).

### 2.4. Micro-CT Analysis

The micro-computed tomography scans were obtained by Skyscan 1172G (Bruker, Kontich, Belgium), a high-resolution 3D imaging system with a L7901-20 Microfocus X-ray Source (Hamamatsu, Shizuoka, Japan).

The digital acquisition of the volumes was obtained with a 0.5 mm Al filter, image pixel/size of 21.96 µm, camera binning 4 × 4, source voltage of 70 kV, source current of 141 µA, and exposure time of 500 ms. The obtained micro-CT volumes of the study samples were acquired and reconstructed by a built-in software package NRecon Skyscan (Version:1.6.6.0; Skyscan Bruker, Allentown, PA, USA).

The three-dimensional reconstruction were obtained by the use of a 3D Visualization Software CTvox v2.5 and DataViewer v1.4.4 (Skyscan Bruker, Allentown, PA, USA) to perform the sample volume rendering and virtual sectioning views. The evaluation of the specimens was provided by the use of the CT-Analyser software v1.13 (OMICRON electronics GmbH, Austria).

### 2.5. Statistical Analysis

The sample size was calculated by a free software available on the website http://clincalc.com/stats/samplesize.aspx, necessary to obtain the number of dental implants necessary for a statistical significance for quantitative analyses of the implant bone contact. A calculation model was approved for dichotomous variables (yes/no effect) by putting the effect incidence designed to caution the reasons, 10% for control implants and 95% for test implants, alpha was set at 0.05 and power at 70%. The optimal sample size for the experimental analysis was a total of six implants for each study group.

The data analysis was determined by the GraphPad Prism 6 software package (GraphPad Software, Inc., San Diego, CA, USA). The Shapiro-Wilks normality test was performed and the BD, BIC, BAIT, and BAOT means were statistically evaluated by the Kruskal Wallis test followed by the Dunn-Bonferroni post hoc. The level of significance for the statistical analysis was set for *p* ≤ 0.05.

## 3. Results

### 3.1. Scanning Electron Microscopy Observations (SEM) and X-ray Spectroscopy Evaluation

The scanning electron microscopy (SEM) analysis showed a nanoporous network structure on the implant surface of both implants. In both of the study groups, the topography of the implant shows the usual microstructure imparted by acid etching.

**Figure 2:**
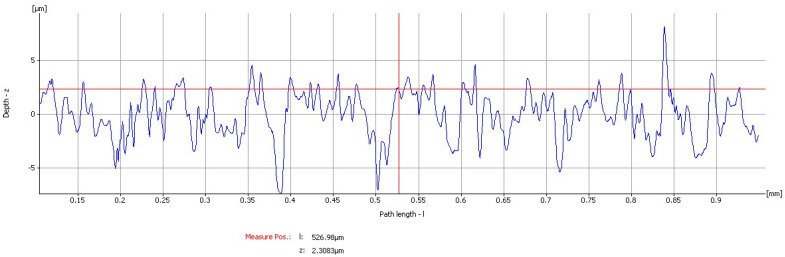
Graphics of the roughness parameters evaluated on the uncoated implant.

**Figure 3:**
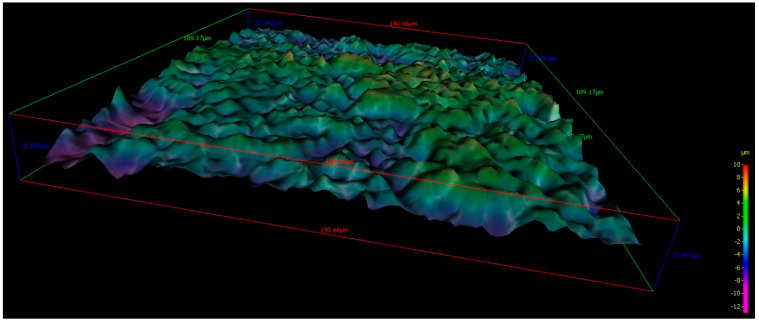
190 × 190 micrometer 3D images of the topographic reconstruction of the implant surface obtained by SEM analysis of an uncoated implant.

The field of view was sufficient to also detect the longer-range microstructure due to sandblasting. For 190 × 190 micrometer areas, the roughness parameter was:

Uncoated Implant: The roughness parameters evaluated gave mean values of mean Ra: 1.729 µm, mean Rq: 2.3501 µm, mean Rt: 15.596 µm, mean Rz: 9.9617 µm, and mean Rmax: 14.865 µm (Figure 2 and Figure 3).

**Figure 4:**
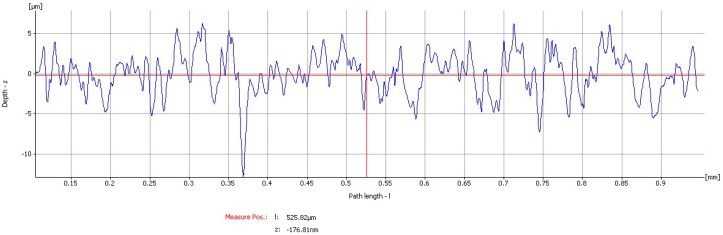
Graphics of the roughness parameters evaluated on the coated implant.

**Figure 5:**
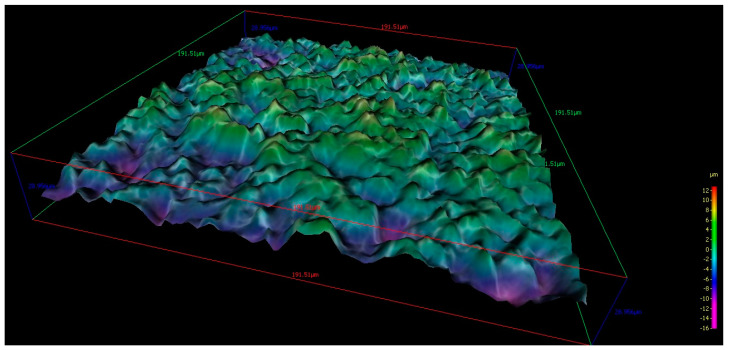
190 × 190 micrometer 3D images of the topographic reconstruction of the implant surface obtained by SEM analysis of a coated implant.

Coated implant: The roughness parameters evaluated gave mean values of mean Ra: 1.7477 µm, mean Rq: 2.2038 µm, mean Rt: 15.834 µm, mean Rz: 10.888 µm, and mean Rmax: 12.43 µm (Figure 4 and Figure 5).

These results confirm that the process does not include the evidence of a deep coating, but of a nanometer-thin surface layer.

### 3.2. Evaluation of the Surface Chemical Composition

The electron superficial spectroscopy for chemical analysis (ESCA) was used on the surface chemical composition use also defined as X-ray photoelectron spectroscopy (XPS, Thermo Fisher Scientific, Waltham, MA, USA). Using the ESCA, the X-ray irradiation hits on the sample surface and the detached energy is measured and analyzed. This method enables to qualitatively and quantitatively determine the elements present in the sample, except for helium (He) and hydrogen (H), and generates a photoelectron spectrum, which includes characteristic peaks for all the elements.

All the elements found at a high concentration in the specimens, such as carbon (C), oxygen (O), and nitrogen (N), were those found in the atmosphere. The concentration of these elements (C, O, and N) decreased in favor of the Ti concentration after sputter cleaning (Table 1). Aluminum (Al) was found in both implant systems according to the sandblasting with an abrasive alumina oxide used for the surface treatment and in the test group Ag^+^ was also found (Table 2).

### 3.3. Micro-CT Evaluation

BD, BIC, BAOT, BAIT, and gaps among the bone and implant were evaluated by means of micrographs.

**Figure 6:**
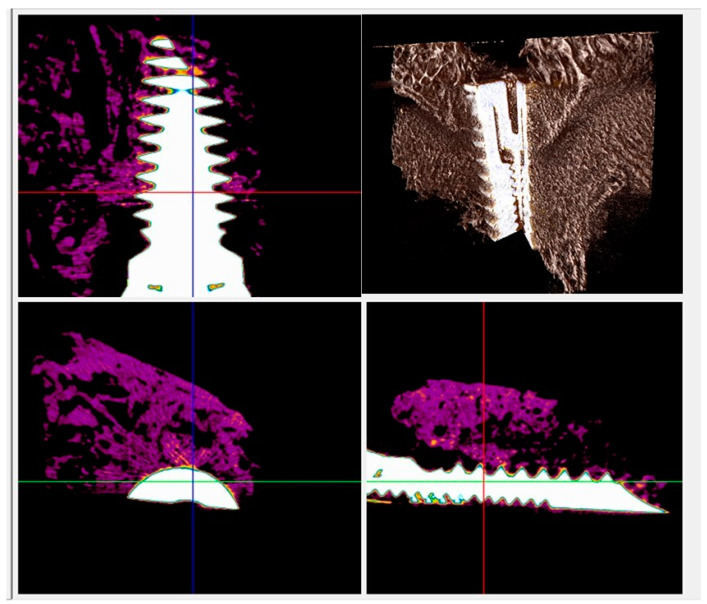
Micro-CT scans taken on the transection planes of the fixture. New bone formation around and in contact with the implant (purple or gray) was evident.

**Figure 7:**
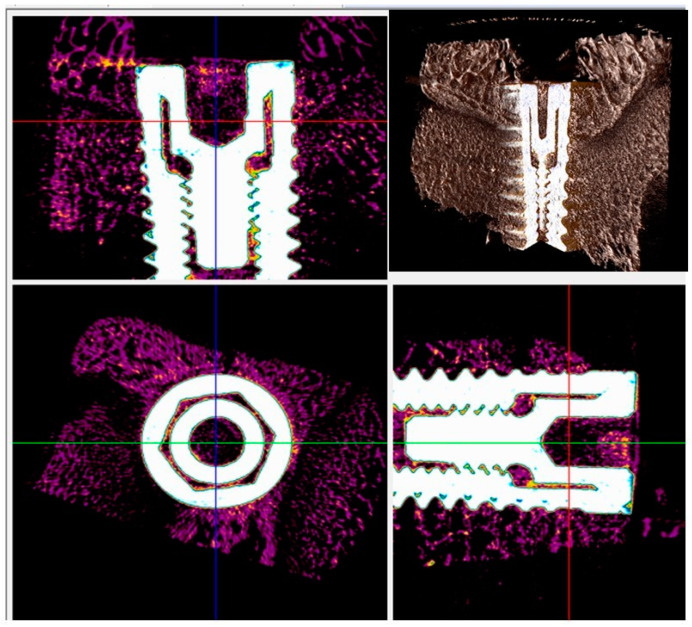
Micro-CT scans for histomorphometric evaluation. The presence of newly formed bone around and in contact with the implant (purple or gray) was found.

In both types of implants, a new bone in direct contact with the surface of the implant was visible in radiographs, and no gaps were detected at 15 days (Figure 5 and Figure 6). At 30 and 60 days the BD, BIC, and BAIT were more present in both implants. No bone resorption inflammation and/or osteolysis were present on either type of surface (Figure 6 and Figure 7).

### 3.4. Histological and Histomorphometrical Results

#### 3.4.1. At Two Weeks

##### Uncoated Implant Surface

**Figure 8:**
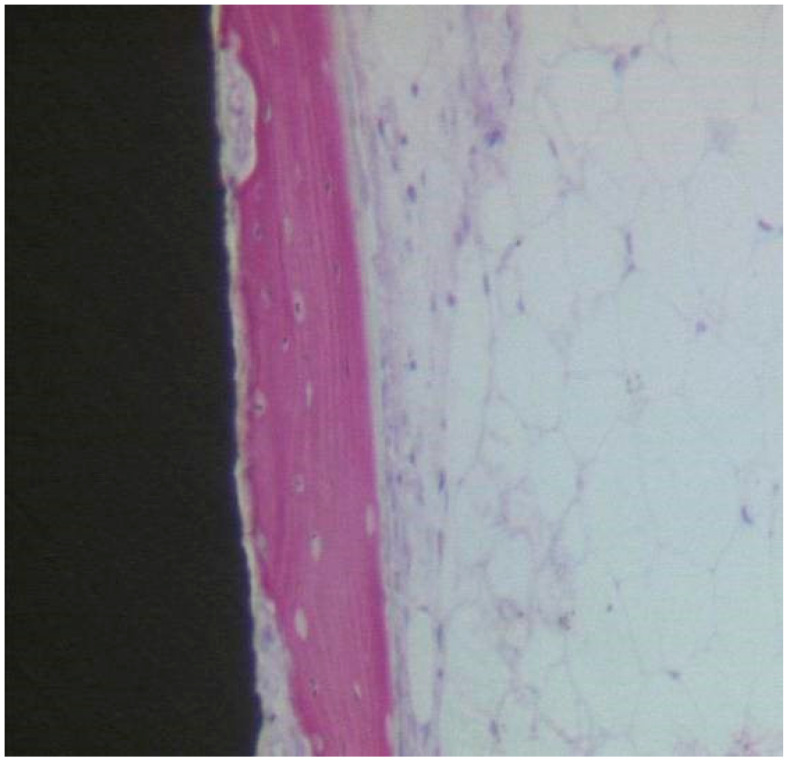
Osteoblasts, depositing osteoid matrix, were observed. Acid fuchsin-toluidine blue 100×.

At low magnification, the specimen’s observation showed aspects of new bone formation in contact with the implant threads (Figure 8). No evidence of multinucleated giant cells or inflammatory cells were evident. The peri-implant bone density (BD) was 16.33 ± 1.1%, while the bone-implant contact percentage (BIC) was 21.67 ± 3.7%, bone area inner threads (BAIT) was 22 ± 0.3%, and bone area outer threads (BAOT) was 20 ± 0.2%.

##### Coated Implant Surface

**Figure 9:**
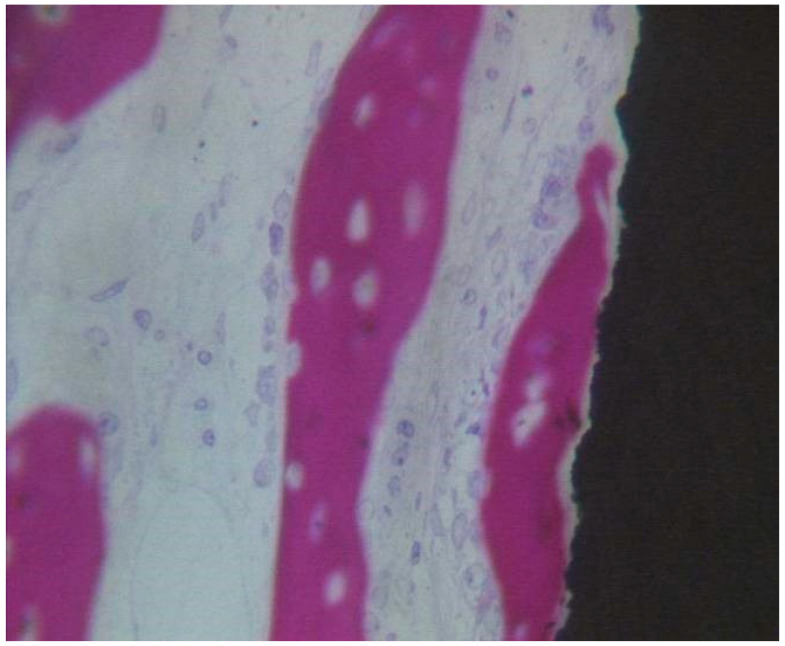
Newly formed bone observed in direct contact to the implant surface. The osteocyte lacunae were large. No evidence of inflammatory cells was present. Many osteoblasts were present, depositing the osteoid matrix. Acid fuchsin-toluidine blue 200×.

In close contact to the dental implant surface there were many newly formed bone trabeculae (Figure 9). Osteoblast cells were observed depositing the osteoid matrix on the implant surface. BD was 15.5 ± 0.9% and BIC was 21.5 ± 2.3%, bone area inner threads (BAIT) was 23 ± 0.5%, and bone area outer threads (BAOT) was 19 ± 0.3%.

#### 3.4.2. At Four Weeks

##### Uncoated Implant Surface

The samples observation showed that aspects of new bone formation were in direct contact with the implant surface, with no evidence of detectable gaps or interposition of fibrous tissue. The bone was growing towards the surface of the implant. BD was 35.33 ± 2.1%, BIC was 34.5 ± 3.2%, bone area inner threads (BAIT) was 27 ± 0.9%, and bone area outer threads (BAOT) was 37 ± 0.2%.

##### Coated Implant Surface

The compact, mature bone was present with smaller marrow spaces. A lesser quantity of osteoblasts was observed. No gaps were present between the bone and the implant surface. No inflammatory cells were observed. BD was 38 ± 1.2%, BIC was 38.83 ± 2.8%, bone area inner threads (BAIT) was 28 ± 0.7%, and bone area outer threads (BAOT) was 35 ± 0.5%.

#### 3.4.3. At Eight Weeks

##### Uncoated Implant Surface

**Figure 10:**
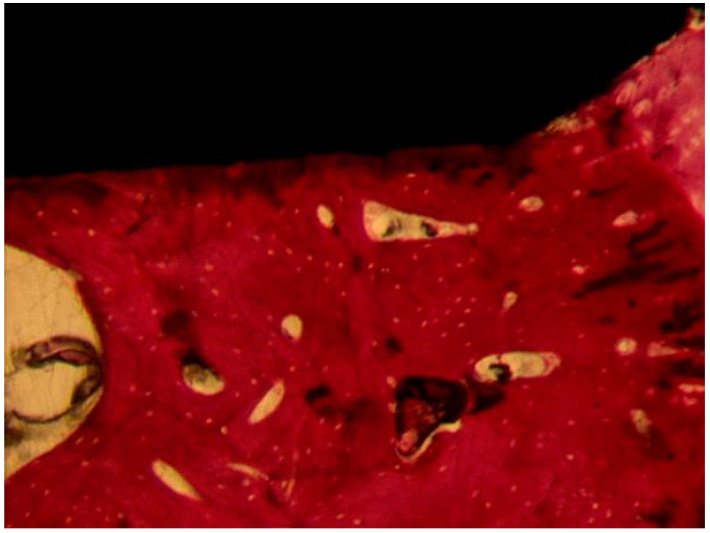
The compact bone was present. No gaps or fibrous connective tissue were present at the interface. Acid fuchsin-toluidine blue 40×.

The compact bone was present (Figure 10). Only a few osteoblasts were present. There were no multinucleated giant cells. No fibrous connective tissue or gaps were present at the bone-implant interface. No inflammatory infiltrate was present. BD was 54 ± 1.6%, BIC was 58.6 ± 3.8%, bone area inner threads (BAIT) was 37 ± 3.1%, and bone area outer threads (BAOT) was 35 ± 1.8%.

##### Coated Implant Surface

**Figure 11:**
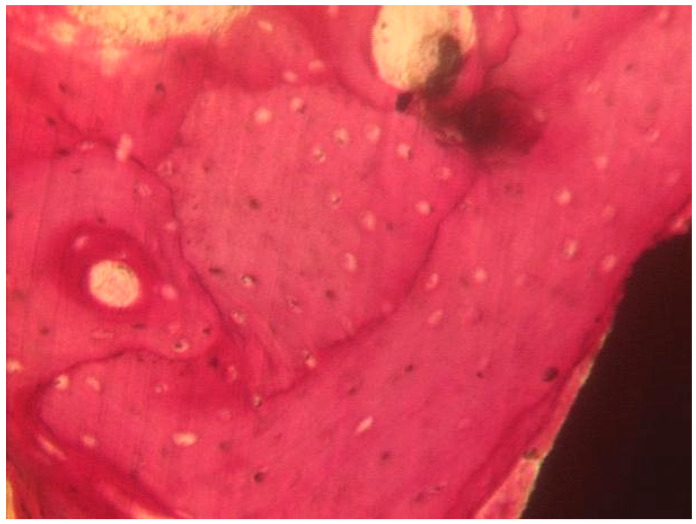
The mature bone was observed at the interface with the implant. Acid fuchsin-toluidine blue 100×.

The mature bone was present (Figure 11). Only in a few areas of the interface, the osteoid matrix not yet mineralized was still present. No inflammatory cells were present. No gaps were present at the bone-implant interface. BD was 56.8 ± 1.4%, BIC was 57.3 ± 2.4%, bone area inner threads (BAIT) was 38 ± 4.2%, and bone area outer threads (BAOT) was 36 ± 3.5%.

#### 3.4.4. Statistical Evaluation

No statistically significant difference was evident in the BD between Control and Test implants at two weeks (*p*-value *=* 0.623), four weeks (*p*-value *=* 0.339), and eight weeks (*p*-value = 0.461). No statistically significant differences were present in the BIC between Control and Test implants at two weeks (*p*-value = 0.938), four weeks (*p*-value = 0.307), and eight weeks (*p*-value = 0.294).

The average and SD values of BIC, BD, BAIT, and BAOT parameters were presented in Table 3. No difference of BIC, BD BAIT, and BAOT ratios at two, four, and eight weeks was evident (*p* > 0.05).

## 4. Discussion

The present results showed that there were no adverse effects on osseointegration, and no statistically significant difference of the bone density and percentages of bone-implant contact between Test and Control implants were evident at all the experimental time points (two, four, and eight weeks). It was hypothesized that dental implants coated with silver ions bound to nano-anatase may not increase the bone in contact with the implant surface, but it is as biocompatible as the uncoated implant surface. No differences were, moreover, present in the roughness measurements (Ra, Rq, Rt, Rz, Rmax) of the two groups. From the results of the present study, it can be deduced that differences detected in implantation tests are not due to the differences of the implant surface topography, although, it highlights the contribution of the different surface chemistries to new bone formation around the implant. The results of the present research support the acceptance of the null hypothesis. Probably, the silver coating does not particularly influence cell movement, proliferation, attachment, and differentiation on the titanium surface without adverse effects. Today, we have a good implant surface that enhances the implant bone contact, however, the peri-implant disease is a common complication of dental implant treatment with an inflammatory reaction, with concomitant loss of the supporting marginal bone. Peri-implantitis and peri-implant mucositis have a high incidence in 18.8 and 63.4% of patients, respectively [21]. For these reasons it is important to have an implant surface that promotes osteogenesis and prevents bacterial adhesion. In fact, the adhesive biofilm at the level of the surfaces connecting the oral ecosystem with the titanium implant is able to produce an influence of the prognosis of the implant life duration [22,23].

The use of the silver coating on the transmucosal portion of the abutment, for example, could be useful in decreasing the quantity of bacteria on the implant surface and, therefore, produce healthier peri-implant tissues [24]. Moreover, such a coating of the implant could be hypothesized, as having clear effects in cases of peri-implant crestal bone loss during peri-implantitis, where it could help in reducing the bacterial charge and help in treating the peri-implant infection. The silver ions and silver salts have low systemic toxicity in vivo [25]. For this reason, they are widely used for application to several medical devices including venous catheters, urinary catheters, wound dressings, and drains. Moreover, titanium oxides have an antibacterial activity. On exposure to air or liquids, a layer of oxide forms on titanium which reduces its reactivity, and this layer interacts with the tissues [13]. Anatase is one of the highly common crystalline forms of TiO_2_, and is normally produced through oxidation of titanium through thermal oxidation or anodization [26,27]. Even if it is irradiated with ultraviolet A light this crystalline form shows a photocatalytic activity [27]. This photocatalytic activity generates many organic compounds [26]. There is a growing interest in the antimicrobial properties of the photocatalytic effects of the titanium dioxide (TiO_2_) [28,29]. In a previous study, we observed that an analogous coating of healing screws produced a lower quantity of bacteria on their surface [30]. Moreover, the addition of the silver nano anatase to composite resins conferred antibacterial properties to adhesives with inhibition of bacterial biofilm and reduction of colony count of S. mutans and S. sanguinis [31,32]. Concerns for the use of this type of coating on the implant surface, due to a possible meddling with the osteoblastic activity can be ruled out by recent results showing that the coating is able to increase the bone implant contact in a rabbit model [33]. The aim of this work was to verify the applicability on titanium implants of antimicrobial coatings based on titanium dioxide functionalized with silver ions. We have not observed an adverse effect with the coated implants, these results are important and will allow us to carry out further comparative future clinical studies.

## 5. Conclusions

The outcomes of the present study showed that the bone healing was similar between the uncoated and silver-anatase coated groups at two, four, and eight weeks after implantation. Histomorphometry analysis showed no significant differences in the bone-implant contact between the two groups without adverse effects. Titanium implants coated with the silver-anatase solution achieved a good osseointegration and did not have an adverse effect on the bone tissue in a rabbit model.

## Figures and Tables

**Table 1:** Series: Characteristic X-ray lines; unn. C [wt%]: The unnormalised concentration in weight percent of the element; norm. C [wt%]: The normalised concentration in weight percent of the element; C Atom [at%]: The atomic weight percent; C Error (1 Sigma) [wt%]: The error in the weight percent concentration at the 1 sigma level.

Element (Atomic Number)	Series	Unn. C [wt%]	norm. C [wt%]	C Atom [at%]	C Error (1 Sigma) [wt%]
C (6)	K-Series	3.71	4.06	13.37	0.75
F (9)	K-Series	2.58	2.83	5.89	0.61
Al (13)	K-Series	5.65	6.19	9.07	0.3
Ti (22)	K-Series	77.37	84.72	69.91	2.17
V (23)	K-Series	2.01	2.2	1.71	0.09

**Table 2:** Series: Characteristic X-ray lines; unn. C [wt%]: The unnormalised concentration in weight percent of the element; norm. C [wt%]: The normalised concentration in weight percent of the element; C Atom [at%]: The atomic weight percent; C Error (1 Sigma) [wt%]: The error in the weight percent concentration at the 1 sigma level.

Element (Atomic Number)	Series	Unn. C [wt%]	norm. C [wt%]	C Atom [at%]	C Error (1 Sigma) [wt%]
C (6)	K-Series	3.61	4.00	12.87	0.66
F (9)	K-Series	1.14	1.27	3.49	0.28
Al (13)	K-Series	2.46	2.73	5.55	0.52
Ti (22)	K-Series	75.18	83.37	67.23	2.11
V (23)	K-Series	1.99	2.21	1.68	0.08
Ag (47)	K-Series	0.09	0.053	0.053	0.002

**Table 3:** Summary of the average and SD of bone area inner threads (BAIT), bone area outer threads (BOAT), bone implant contact (BIC), and bone density (BD). Values were measured by the Micro-CT analysis.

		BIC	*p*-Value	BD	*p*-Value	BAIT	*p*-Value	BAOT	*p*-Value
**Two weeks**	Uncoated Surface	21.67 ± 3.7	*p* = 0.938	16.33 ± 1.1	*p* = 0.623	22 ± 0.3	*p* = 0.2581	20 ± 0.2	*p* = 0.2138
	Coated	21.5 ± 2.3		15.5 ± 0.9		23 ± 0.5		19 ± 0.3	
**Four weeks**	Uncoated Surface	34.5 ± 3.2	*p* = 0.307	35.33 ± 2.1	*p* = 0.339	27 ± 0.9	*p* = 0.8739	37 ± 0.2	*p* = 0.1060
	Coated	38.83 ± 2.8		38 ± 1.2		28 ± 0.7		35 ± 0.5	
**Eight weeks**	Uncoated Surface	58.6 ± 3.8	*p* = 0.294	54 ± 1.6	*p* = 0.461	37 ± 3.1	*p* = 0.7194	35 ± 1.8	*p* = 0.1571
	Coated	57.3 ± 2.4		56.8 ± 1.4		38 ± 4.2		36 ± 3.5	
